# Multicenter inception cohort of enthesitis-related arthritis: variation in disease characteristics and treatment approaches

**DOI:** 10.1186/s13075-017-1297-x

**Published:** 2017-05-02

**Authors:** Sabrina Gmuca, Rui Xiao, Timothy G. Brandon, Ilaria Pagnini, Tracey B. Wright, Timothy Beukelman, Esi M. Morgan, Pamela F. Weiss

**Affiliations:** 1 Division of Rheumatology, Department of Pediatrics, The Children’s Hospital of Philadelphia, Philadelphia, PA USA; 2 Department of Biostatistics and Epidemiology, Perelman School of Medicine, University of Pennsylvania, Philadelphia, PA USA; 3 University of Florence and Anna Meyer Children’s Hospital, Florence, Italy; 4 University of Texas Southwestern Medical Center and Texas Scottish Rite Hospital of Children, Dallas, TX USA; 5 University of Alabama at Birmingham, Birmingham, AL USA; 6 Cincinnati Children’s Hospital, Cincinnati, OH USA; 7 University of Pennsylvania Perelman School of Medicine and Children’s Hospital of Philadelphia, Philadelphia, PA USA; 8 Department of Pediatric Rheumatology, The Children’s Hospital of Philadelphia, 34th Street and Civic Center Boulevard, Wood Building, Fourth Floor, Philadelphia, PA 19104 USA

**Keywords:** Enthesitis-related arthritis, Juvenile idiopathic arthritis, HLA-B27, Classification criteria

## Abstract

**Background:**

Enthesitis-related arthritis (ERA) is a specific subtype of juvenile idiopathic arthritis (JIA) defined according to the International League of Associations for Rheumatology (ILAR) criteria. We aimed to characterize the clinical features and treatment regimens in an inception cohort of children with ERA.

**Methods:**

We performed a retrospective, cross-sectional, multicenter cohort study including subjects diagnosed with ERA between 1989 and 2012. Patients all fulfilled the ILAR criteria for ERA within 3 months of initial presentation to the rheumatology clinic. Differences in the prevalence of clinical criteria across study sites and by human leukocyte antigen (HLA)-B27 status were assessed using the Wilcoxon rank-sum or chi-square test, as appropriate.

**Results:**

Two hundred thirty-four children met the inclusion criteria. Their median age at diagnosis was 11.6 years, and 59% were HLA-B27-positive. Sixty-nine percent had enthesitis and arthritis at the time of diagnosis. Seventy-eight percent had a pauciarticular onset. The prevalence of all ILAR criteria at diagnosis, except arthritis and acute anterior uveitis, differed significantly across sites (all *p* < 0.01). Medication use varied significantly across sites for children with peripheral arthritis (*p* < 0.001), but not for sacroiliitis or enthesitis only. Nonsteroidal anti-inflammatory drugs and disease-modifying antirheumatic drugs were the most commonly prescribed treatments, with anti-TNF agents primarily being initiation for sacroiliitis. HLA-B27 positivity was associated with male sex, higher active joint count, sacroiliitis, and higher disease activity at disease onset.

**Conclusions:**

The majority of children had a pauciarticular onset, and several statistically significant clinical differences based on HLA-B27 status were identified. The observed heterogeneity in clinical presentation across sites reflects either true differences in patient populations or differences in how the ILAR criteria are being applied.

## Background

Juvenile idiopathic arthritis (JIA) is the most common pediatric rheumatic disease [1] and includes a number of heterogeneous arthritides. JIA is classified into seven mutually exclusive categories by the International League of Associations of Rheumatology (ILAR) classification [2]. Enthesitis-related arthritis (ERA) accounts for approximately 10% to 20% of JIA cases [3, 4] and, as alluded to by its name, is distinguished mainly by involvement of the entheses (where tendons and ligaments insert into bone). A significant proportion of current knowledge regarding ERA is based on data from adults with ankylosing spondylitis and spondyloarthritis (SpA), with a paucity of studies specifically focused on the ERA population. Additionally, application of adult data to children with ERA is not ideal, as evidenced by recognized clinical differences between the two populations, including less severe spinal involvement and more pronounced enthesitis and peripheral arthritis in children [5, 6]. Therefore, there is a critical need for research focused specifically on the ERA population.

The majority of research conducted in children with ERA has been in the context of juvenile SpA, which includes other conditions such as inflammatory bowel disease and psoriatic arthritis. Epidemiologic studies focused on children with ERA as a distinct cohort are scarce. The largest study to date included 312 ERA subjects from a single center in northern India [7]. In this study, the mean age was 11.3 years, there was a male predominance (9:1), and 87% were human leukocyte antigen (HLA)-B27-positive. The high HLA-B27 positivity and single-center nature of the study make its applicability to other populations uncertain. Other studies focused on this JIA category have limited sample sizes ranging from 19 to 84 subjects [8–12]. Additionally, few studies have examined treatment practices for this group of children. Treatment recommendations have been published by the American College of Rheumatology (ACR) and are based primarily on the number of active joints and the presence of sacroiliitis [13]. It is unknown whether routine practice reflects these recommendations.

The significance of HLA-B27 positivity in this population has also not been well characterized. In children classified as having ERA on the basis of ILAR criteria, the proportion of HLA-B27 positivity ranges from 45% to 88% in published studies [6, 7, 9, 10, 12]. HLA-B27 positivity in this group of children also seems to vary by geographic location. Cohorts from India and Mexico have higher reported prevalence of HLA-B27 positivity than North American and European cohorts [7, 14]. The importance, or lack thereof, of HLA-B27 in shaping the clinical phenotype and course of these patients remains unclear. Both acute anterior uveitis (AAU) and sacroiliitis have been reported to have an association with HLA-B27 [15, 16]. Researchers in prior studies have also reported HLA-B27 as an indicator of a poor prognosis among all categories of JIA, with HLA-B27-positive patients having more inflammatory back pain [17], greater disease activity [18], and decreased likelihood of remission after 8 years of disease [19] than children with JIA who are HLA-B27-negative.

We performed a retrospective, cross-sectional, multicenter cohort study that included subjects from five pediatric rheumatology centers (in North America and Europe) to evaluate the clinical presentation and treatment regimens of children with ERA. We also aimed to test whether children classified as having ERA are phenotypically distinct based on HLA-B27 positivity, which might have implications for the assessment or management of these two subgroups of ERA.

## Methods

The protocol for this study was reviewed and approved by each institution’s committee for the protection of human subjects.

### Study site and participants

We performed a retrospective cross-sectional study using a multicenter database of children with ERA from five pediatric rheumatology centers. The study sites were Children’s Hospital of Philadelphia (Philadelphia, PA, USA), Children’s of Alabama (Birmingham, AL, USA), Cincinnati Children’s Hospital Medical Center (Cincinnati, OH, USA), Texas Scottish Rite Hospital for Children (Dallas, TX, USA), and Meyer Children’s Hospital (Florence, Italy). All subjects met the following inclusion criteria: (1) diagnosis of ERA according to the ILAR criteria (Table 1) within 3 months of initial presentation to rheumatology clinic with signs or symptoms of juvenile arthritis, (2) aged ≤16 years at symptom onset, and (3) evaluated at one of the study sites during the study period (1989–2012). Chart reviewers independently applied the ILAR criteria to all study subjects to establish the diagnosis of ERA, given that this nomenclature did not exist for the years prior to 1995. Eligibility criteria were verified by the coordinating center (Children’s Hospital of Philadelphia); 85 children were excluded after this process.

**Table 1:** International League of Associations for Rheumatology criteria for enthesitis-related arthritis

Inclusion criteria	Exclusion criteria
Arthritis and enthesitis	▪ Psoriasis or a history of psoriasis in the patient or a first degree relative
*or*	▪ Presence of IgM RF on at least two occasions at least 3 months apart
Arthritis *or* enthesitis with at least two of the following:	▪ Systemic JIA in the patient
▪ Sacroiliac joint tenderness and/or inflammatory spinal pain ▪ Presence of HLA-B27 ▪ Onset of arthritis in a male over 6 years of age ▪ Family history in at least one first degree relative of ankylosing spondylitis, ERA, sacroiliitis with IBD, reactive arthritis or acute anterior uveitis ▪ Acute anterior uveitis	

*Abbreviations: ERA* Enthesitis-related arthritis, *HLA* Human leukocyte antigen, *IBD* Inflammatory bowel disease, *IgM* Immunoglobulin M, *RF* Rheumatoid factor, *JIA* Juvenile idiopathic arthritis

### Clinical characteristics

The baseline visit was defined as the first rheumatology appointment at which the patient exhibited clinical signs or symptoms of JIA (enthesitis, arthritis, AAU, or inflammatory back pain), regardless of whether the child was classified as having ERA at that time. Patient data were abstracted from the medical charts and included the following: demographics, family history, clinical features, patient-reported outcomes, and medications prescribed. Peripheral arthritis was defined as joint swelling or, in the absence of swelling, limited range of motion plus tenderness [20, 21]. Enthesitis was defined as tenderness to palpation according to the ILAR criteria [2]. Because there is no standardized pediatric examination of the entheses, the sites routinely examined at each study site varied. The entheses insertion sites routinely examined included interosseous ligaments of the sacroiliac joint (all sites), hip flexors at the greater trochanter (four sites), quadriceps at the patella (all sites), infrapatellar tendon at the patella (all sites), infrapatellar tendon at the tibial tubercle (four sites), Achilles tendon (all sites), and plantar fascia at calcaneus (all sites). Sacroiliitis was defined as evidence of inflammation (e.g., bone marrow edema, joint space enhancement, or erosions/sclerosis) visualized by magnetic resonance imaging (MRI) [22, 23]. Ascertained laboratory values included the C-reactive protein (CRP), erythrocyte sedimentation rate (ESR), antinuclear antibody (ANA), and HLA-B27.

Patient-reported outcomes included patient-reported pain over the past week using a visual analogue scale (range 0–10); the Childhood Health Assessment Questionnaire (CHAQ), which is 30-item questionnaire that assesses 8 domains of physical function with scores ranging from 0 to 3 [24, 25]; and patient global assessment of disease activity using a visual analogue scale (range 0–10). CHAQ scores of 0.13, 0.63, and 1.75 represent mild, mild to moderate, and moderate impairment, respectively [26]. Physician global assessment of disease activity (physician global) was also abstracted (range 0–10) [27, 28]. Two composite disease activity scores were calculated: the clinical Juvenile Arthritis Disease Activity Score (cJADAS) (comprised of the active joint count, physician global evaluation, and parent global evaluation) and the Juvenile Spondyloarthritis Disease Activity Index (JSpADA). The cJADAS was developed for use in all categories of JIA, with a score ranging from 0 to 30, where higher scores indicate more active disease [29]. Cutoffs for minimal and high disease activity using the cJADAS are <1.5 and >4, respectively, in oligoarticular disease and <2.5 and >8.5, respectively, in polyarticular disease [30]. The JSpADA was developed specifically for use in juvenile SpA and consists of eight items (arthritis, enthesitis, patient pain assessment, inflammatory markers, morning stiffness, clinical sacroiliitis, AAU, and back mobility). Scores range from 0 to 8, with higher scores being indicative of more active disease [31].

Because this study involved retrospective data collection, there were missing data. Missing data for laboratory values (ANA, HLA-B27, ESR, or CRP) did not exceed 13%. Patient-reported outcomes were missing for approximately 50% of visits; missing values were not imputed. Age, sex, and active joint count did not significantly differ between those visits with and without missing patient-reported outcomes (all *p* > 0.05) or between those with and without calculable cJADAS and JSpADA scores (*p* > 0.05).

For evaluation of treatments, children were considered to belong to one of the following three mutually exclusive phenotypes with the following hierarchy: (1) sacroiliitis (MRI-defined), (2) peripheral arthritis, and (3) enthesitis only. Treatments evaluated included nonsteroidal anti-inflammatory drugs (NSAIDs), intra-articular (IA) glucocorticoid injections, disease-modifying antirheumatic drugs (DMARDs: methotrexate, leflunomide, sulfasalazine), and biologic therapy with tumor necrosis factor (TNF)-α blockade (adalimumab, etanercept, infliximab). For comparison of clinical features between children based on HLA-B27 status, the cohort was restricted to those who had both arthritis and enthesitis. We reasoned that if we were to compare those who were HLA-B27-positive with those who were HLA-B27-negative without restricting the analyses to those with both arthritis and enthesitis, we might be detecting differences based on the classification criteria met rather than true differences between these groups based on HLA-B27 status.

### Analysis

Subject characteristics were summarized by median and interquartile range (IQR) for continuous variables and frequency and percentage for categorical variables. Differences in the prevalence of clinical criteria and medication use for clinical phenotypes across study sites were assessed by the chi-square test. Differences in clinical characteristics by HLA-B27 status were assessed using the Wilcoxon rank-sum or chi-square test, as appropriate. *P* values less than 0.05 were considered statistically significant. All analyses were performed using Stata release 14.2 statistical software (StataCorp, College Station, TX, USA).

## Results

### Clinical characteristics

Two hundred thirty-four children and adolescents met the study inclusion criteria. Table 2 shows the demographic and clinical characteristics of this cohort at diagnosis. Children and adolescents with ERA were predominantly male (72.2%) and Caucasian (82.9%). The median age at diagnosis was 11.6 years (IQR 9.8–13.7). One hundred thirty-five (59.2%) children were HLA-B27-positive. The median number of active joints was 2 (IQR 1–4), and the median number of tender entheses was 2 (IQR 1–4). The most commonly affected joints were the knee (107 patients with at least one affected knee [45.7%]), ankle (*n* = 84 patients [35.9%]), wrist (*n* = 46 [19.7%]), and hip (*n* = 45 [19.2%]). Seventy-eight percent of children had a pauciarticular onset (fewer than five joints). The most frequently tender entheses were the insertions of the infrapatellar tendon on the patella (*n* = 103 patients [44%]), Achilles tendon (*n* = 77 [32.9%]), and the interosseous ligaments of the sacroiliac joint (*n* = 71 [30.3%]).

**Table 2:** Patient characteristics at time of enthesitis-related arthritis classification

	All subjects (*n* = 234)
Demographics, median (IQR) or *n* (%)	
Age in years	11.6 (9.8–13.7)
Sex, male	169 (72.2)
Race, Caucasian	194 (82.9)
Family history of HLA-B27-associated disease	36 (15.4)
Clinical characteristics, median (IQR) or *n* (%)	
HLA-B27 positivity	135 (59.2)
Active joint count	2 (1–4)
Tender entheses count	2 (1–4)
MRI-defined sacroiliitis (*n* = 45 with imaging)	25 (55.6)
Polyarticular onset	52 (22.2)
ANA-positive	49 (23.4)
CRP, abnormal/elevated	39 (26.9)
ESR, abnormal/elevated	93 (45.8)
Patient-/physician-reported outcomes, median (IQR)	
Physician global disease activity assessment (0–10)	2.5 (2–4)
Juvenile Spondyloarthritis Disease Activity Index (JSpADA) (0–8)	3 (2.5–4)
Juvenile Arthritis Disease Activity Score (cJADAS), excluding ESR (0–30)	9 (5–14)
Patient/parent global disease activity assessment (0–10)	4 (2–6)
Function (CHAQ) (0–3)	0.563 (0–1)
Patient/parent pain assessment (0–10)	4 (2–7)

*Abbreviations*: *IQR* Interquartile range, *HLA* Human leukocyte antigen, *MRI* Magnetic resonance imaging, *ANA* Antinuclear antibody, *CRP* C-reactive protein, *ESR* Erythrocyte sedimentation rate, *CHAQ* Childhood Health Assessment Questionnaire

Denominators for clinical characteristics varied because of missing data: HLA-B27 status (*n* = 228), magnetic resonance imaging-defined sacroiliitis (*n* = 45), ANA (*n* = 209), CRP (*n* = 145), ESR (*n* = 203), physician global disease activity assessment (*n* = 126), JSpADA (*n* = 116), patient/parent global disease activity assessment (*n* = 102), CHAQ (*n* = 120), patient/parent pain assessment (*n* = 129), cJADAS (JADAS3–10) (*n* = 86)

**Fig. 1:**
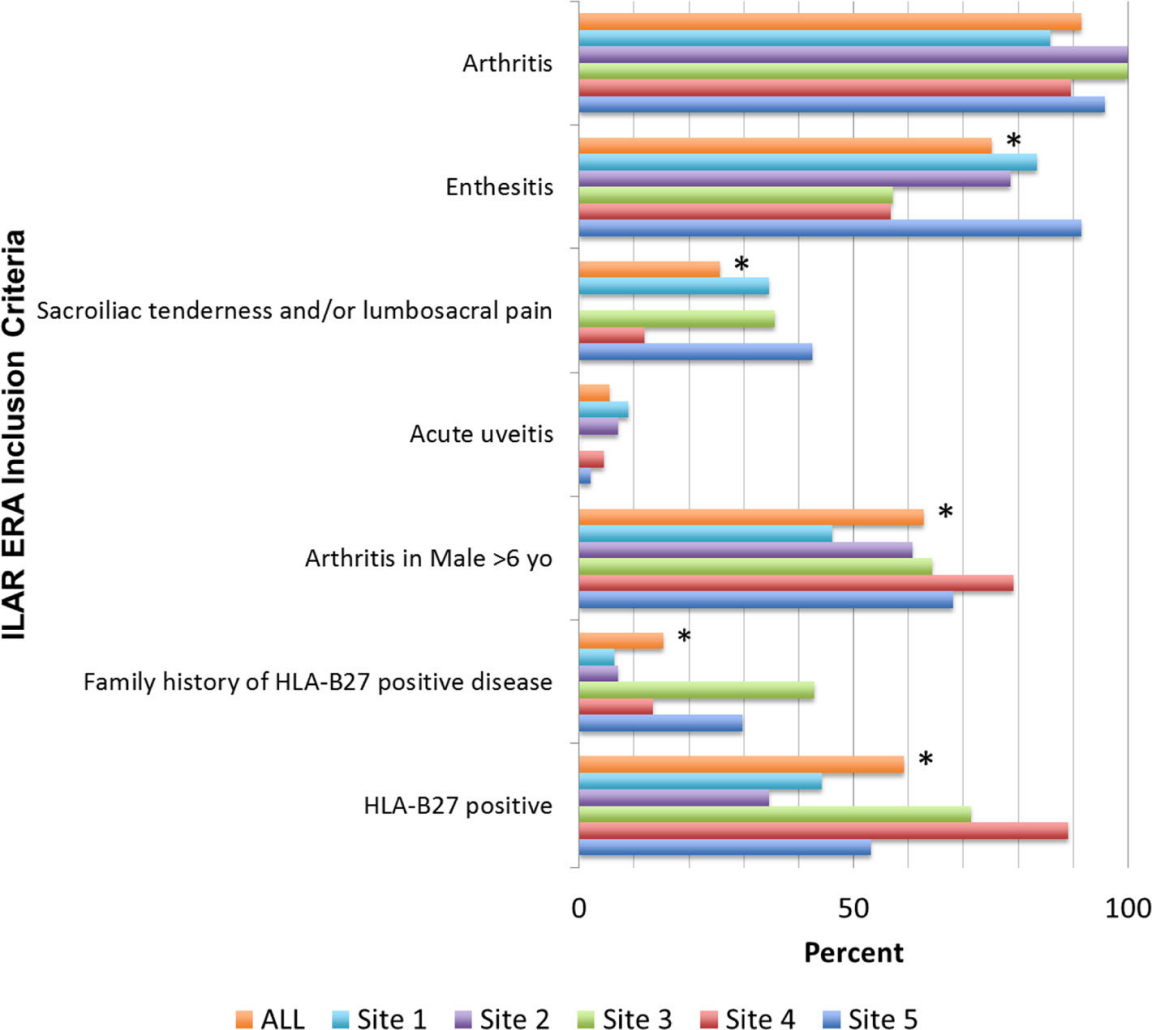
International League of Associations for Rheumatology (ILAR) enthesitis-related arthritis (ERA) inclusion criteria by enrollment site. * *p* < 0.05 for between-group comparisons. *HLA* Human leukocyte antigen

Figure 1 shows the percentage of children who met each of the ILAR ERA criteria. The majority of patients met the ILAR criteria for a diagnosis of ERA at the first rheumatology visit (*n* = 214 [91.5%]), with 18 subjects (7.7%) meeting the criteria at the second visit and 2 subjects (0.9%) meeting the criteria by the third visit. One hundred sixty-two (69.2%) children had arthritis *and* enthesitis, and 72 (30.8%) children had arthritis *or* enthesitis plus at least two additional ILAR criteria. Of the 72 children presenting with arthritis or enthesitis plus at least two additional ILAR criteria, 55 had arthritis only and 17 had enthesitis only. Because arthritis (91.5%) and enthesitis (75.2%) are requirements under the ILAR criteria for ERA, it was not surprising that they were the two most common disease manifestations followed by the presence of arthritis in a male over the age of 6 years (62.8%). Sacroiliac tenderness was present in 25.6% (*n* = 60) of patients, and AAU was present in only 5.6% (*n* = 13) of patients. Although more than half (59.2%) of all patients were HLA-B27-positive, this proportion varied significantly across sites, ranging from 35% to 89% (*p* < 0.001) (Fig. 1). The prevalence of arthritis and AAU did not differ significantly across sites. The prevalence of each of the remaining ILAR criteria, however, was also significantly different across sites (Fig. 1) (all *p* < 0.01): enthesitis (range: 57% to 91%), sacroiliac tenderness or inflammatory lumbosacral pain (range: 12% to 43%), onset of arthritis in a male over 6 years (range: 46% to 79%), and history of HLA-B27-associated disease in a first-degree relative (range: 6% to 43%).

Table 2 lists patient- and physician-reported disease activity assessments. Disease activity at diagnosis was moderate as assessed globally by patients/parents (median 4, IQR 2–6) and physicians (median 2.5, IQR 2–4). Patients also had moderate disease activity as evaluated by composite disease activity measures (JSpADA median 3, IQR 2.5–4; cJADAS median 9, IQR 5–14). Patients reported moderate pain (median score of 4, IQR 2–7) and between mild and mild to moderate disability as assessed by the CHAQ (median score of 0.563, IQR 0–1). Twenty-seven percent (*n* = 39) had systemic inflammation as evidenced by an elevated CRP level, and 45.8% (*n* = 93) had an elevated ESR (Table 2).

### Medication use

**Fig. 2:**
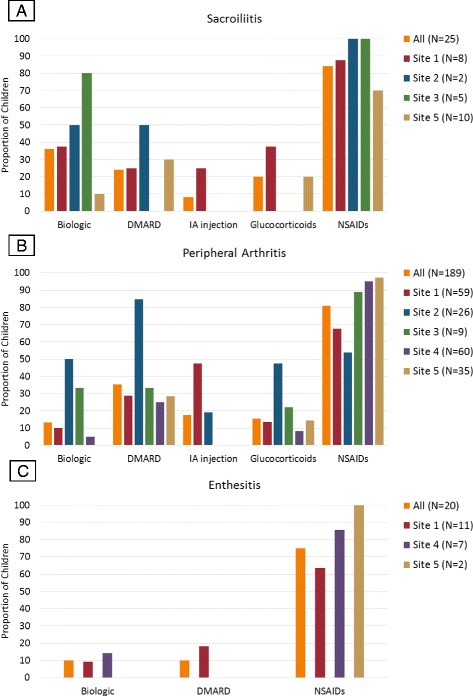
Medications prescribed based on clinical phenotype. Medications prescribed across sites for (**a**) sacroiliitis (magnetic resonance imaging [MRI]-defined), (**b**) peripheral arthritis, and (**c**) enthesitis only. Children were considered as having one of the following three mutually exclusive phenotypes with the following hierarchy: (1) sacroiliitis (MRI-defined), (2) peripheral arthritis, and (3) enthesitis. Biologic medications included tumor necrosis factor-α inhibitors (adalimumab, etanercept, infliximab). Sacroiliitis data were not available for site 4. No children from site 2 or 3 were diagnosed with isolated enthesitis, and of those with enthesitis, none were prescribed IA injections or glucocorticoids. *DMARD* Disease-modifying antirheumatic drug, *Glucocorticoids* Oral glucocorticoids, *IA* Intra-articular glucocorticoid injection, NSAIDs Nonsteroidal anti-inflammatory drugs

Figure 2 demonstrates unadjusted medication use at diagnosis based on clinical phenotype. Overall, NSAIDs were the most commonly prescribed medication class (*n* = 189 [80.8%]), followed by DMARDs (*n* = 75 [32.1%]). Oral glucocorticoids (14.6%), IA glucocorticoid injections (15.0%), and biologic agents (15.4%) were less commonly used. Medication use varied significantly among sites for children with peripheral arthritis (*p* < 0.001) but not for those with sacroiliitis or enthesitis only. NSAIDs-only was the most prevalent treatment across every disease phenotype. Children presenting with MRI-defined sacroiliitis were prescribed NSAIDs only (40%), biologic monotherapy (32%), or a combination of a biologic and a DMARD (4%). Children with arthritis at diagnosis were most commonly prescribed NSAIDs only (43%) or DMARD monotherapy (27%). Subjects with only enthesitis at diagnosis were treated primarily with NSAIDs (82%). IA glucocorticoid injections were reserved for children with peripheral arthritis (11.3%). Oral corticosteroids were prescribed for those with sacroiliitis (4.0%) and peripheral arthritis (5.9%).

### HLA-B27 status and clinical phenotype

In order to determine whether patient demographics, clinical characteristics, or ILAR criteria fulfillment differed on the basis of HLA-B27 status, we restricted our analyses to patients who met ILAR criteria by having both arthritis *and* enthesitis (*n* = 156). Clinical characteristics of HLA-B27-positive versus HLA-B27-negative children are presented in Table 3. Among this restricted cohort, 44% (*n* = 69) were HLA-B27-positive. As in the primary analysis, male sex and Caucasian racial predominance persisted.

**Table 3:** Clinical features by human leukocyte antigen B27 status

	All^a^ (*n* = 156)	HLA-B27 positive (*n* = 69)	HLA-B27 negative (*n* = 87)	*p* Value
Demographics and clinical findings, *n* (%) or median (IQR)				
Age in years	11.5 (9.6, 13.6)	12.3 (10.3, 14.1)	11.0 (8.8,13.2)	*<0.01*
Sex, male	100 (64.1)	50 (72.5)	50 (57.5)	0.05
Race, Caucasian	129 (82.7)	59 (85.5)	70 (80.5)	0.66
Active joint count	2 (1–4)	3 (2–5)	2 (1–4)	*<0.01*
Tender entheses count	2 (2–4)	2 (1–3)	2 (2–5)	*0.01*
MRI-defined sacroiliitis	22 (57.9)	15 (83.3)	7 (35.0)	*<0.01*
Polyarticular onset	36 (23.1)	20 (29.0)	16 (18.4)	0.15
ANA-positive	31 (21.4)	10 (15.6)	21 (25.9)	0.13
CRP, abnormal/elevated	31 (28.4)	13 (31.0)	18 (26.9)	0.65
ESR, abnormal/elevated	57 (41.9)	34 (54.8)	23 (31.1)	*<0.01*
Outcome measures, median (IQR)				
Physician disease activity (0–10)	2.4 (2–3)	2 (2–3)	2.6 (2–4)	0.07
Juvenile Spondyloarthritis Disease Activity Index (JSpADA) (0–8)	3 (2.5–4)	3.5 (2.5–4)	3 (2.5–3.5)	*0.04*
Patient/parent disease activity (0–10)	4 (2–6)	4 (2–5)	4 (2–6)	0.63
Function (CHAQ) (0–3)	0.625 (0–1)	0.375 (0–1)	0.625 (0–1)	0.85
Patient/parent pain (0–10)	4 (2–7)	4 (3–7)	4 (2–7)	0.71
Juvenile Arthritis Disease Activity Score, excluding ESR (cJADAS) (0–30)	9 (5–13)	9 (5–13)	9 (5–14)	0.98
ILAR criteria, *n* (%)				
Acute, symptomatic uveitis	7 (4.5)	4 (5.8)	3 (3.5)	0.48
Arthritis in a male over 6 years of age	93 (59.6)	50 (72.5)	43 (49.4)	*<0.01*
Family history of HLA-B27-associated disease	18 (11.5)	7 (10.1)	11 (12.6)	0.63
Sacroiliac tenderness or lumbosacral pain	46 (29.5)	20 (29.0)	26 (29.9)	0.9

*Abbreviations:* HLA-B27 Human leukocyte antigen B27, *MRI* Magnetic resonance imaging, *ANA* Antinuclear antibody, *CRP* C-reactive protein, *ESR* Erythrocyte sedimentation rate, *IQR* Interquartile range, *CHAQ* Childhood Health Assessment Questionnaire

Analyses restricted to patients who had both arthritis and enthesitis at the time of diagnosis. Denominators varied due to missing data: MRI-defined sacroiliitis (*n* = 38); ANA (*n* = 145); CRP (*n* = 109); ESR (*n* = 136); Physician disease activity (*n* = 97); JSpADA (*n* = 92); Patient/parent disease activity (*n* = 79); CHAQ (*n* = 92); Patient/parent pain (*n* = 100); cJADAS (JADAS3–10) (*n* = 69)

^a^ Six subjects who had arthritis and enthesitis at diagnosis were excluded because they were missing HLA-B27 status. *P*-values less than 0.05 are in italics and considered clinically significant

There was an older median age of onset in the HLA-B27-positive children (12.3 versus 11.0 years; *p* = 0.01). In comparison with HLA-B27-negative children, HLA-B27-positive children had a higher active joint count (*p* < 0.01) and were more likely to have sacroiliitis (*p* < 0.01). A greater proportion of HLA-B27-positive children had an elevated ESR (54.8% versus 31.1%; *p* < 0.01). The JSpADA was significantly higher in those who were HLA-B27-positive than in those who were HLA-B27-negative (*p* = 0.04), whereas there was no significant difference in the cJADAS or physician global disease activity assessment. Those who were HLA-B27-positive were more likely to develop arthritis after the age of 6 years (*p* < 0.01). There were no other differences in the ILAR criteria based on HLA-B27 status.

## Discussion

To our knowledge, this study comprises the largest multicenter international inception cohort of children with ERA to date. Children with ERA were predominantly male, Caucasian, and HLA-B27-positive; these findings are in accordance with previously published cohorts [9, 32]. An overwhelming majority of patients (91.5%) met the ILAR criteria for a diagnosis of ERA at their first visit to a pediatric rheumatologist. The majority of subjects had both arthritis and enthesitis at the time of diagnosis. Most children had a pauciarticular onset of disease, and the most commonly affected joints were the knees, ankles, wrists, and hips. Enthesitis was most common at the attachments of the infrapatellar tendon on the inferior pole of the patella, the interosseous ligaments of the sacroiliac joints, and the Achilles tendon. These findings highlight the importance of carefully assessing for arthritis and enthesitis in all possible locations when examining a new patient presenting with concern for JIA. Perhaps special attention should be paid to the aforementioned anatomical sites when considering a diagnosis of ERA. NSAIDs and DMARDs were the most often prescribed first-line therapies. There was increased use of biologic agents, however, for the treatment of children with sacroiliitis. Systemic glucocorticoids were reserved for children with arthritis or sacroiliitis. Children who were HLA-B27-positive tended to be older and male, with a higher active joint count, more sacroiliitis, and higher disease activity.

Several findings warrant additional discussion. First, we found significant variability in the presenting features and initial treatment strategies between sites. The variation in presenting features may reflect either true differences by geographic location or differences between sites in the assessment of particular features. There are no standardized definitions for what constitutes positive enthesitis. The entheses examined and thresholds for declaring positive findings varied not only between sites but also between physicians at each site. Variation in family history could reflect recall bias, differences in how family history was recorded at each site, or true variation. In terms of the initial treatment of ERA, we found that NSAIDs and DMARDs were most often prescribed. We did note, however, that there was increased use of biologic agents for the treatment of children with sacroiliitis, and IA glucocorticoids were reserved for cases of peripheral arthritis. Despite the intersite variation in treatments, our findings suggest that pediatric rheumatologists are generally adhering to ACR-recommended treatment guidelines [13, 33, 34] to initiate a TNF-α blocker for axial disease, especially early in the disease course [35]; however, barriers imposed by insurance companies may account for the fact that only 15% of patients received biologic agents in the first 3 months of disease. Because many insurance companies require that patients first fail methotrexate before they will provide coverage for a biologic agent, this logistical issue may be driving our findings. These differences highlight the need for standardized assessments for ERA as well as comparative effectiveness studies of the different treatment options.

Second, this study demonstrates a few statistically significant clinical differences based on HLA-B27 status. Children who were HLA-B27-positive tended to be older and male, with a higher active joint count and more sacroiliitis. We restricted our analyses of HLA-B27-positive versus HLA-B27-negative patients to children with both arthritis and enthesitis because of a concern for selection bias owing to the inherent nature of the minor inclusion classification criteria. Doing so strengthened our findings by removing any reliance on criteria selecting for HLA-B27 status, sex, and age. HLA-B27-positive children also had a higher disease activity as assessed by the JSpADA. Surprisingly, in contrast to adult studies, we did not find an association between HLA-B27 status and the presence of AAU [15, 36]. However, in a recent study of patients with ERA in which researchers enrolled subjects prospectively over a 3-year period an overall prevalence of AAU of 15.4% was found with no statistically significant difference based on HLA-B27 status [7]. This suggests that there may be additional phenotypic differences between child- and adult-onset diseases. Alternatively, one might theorize that AAU manifests later in the disease course of ERA and is not a frequent symptom at the time of initial presentation. Because this was a cross-sectional study at the time of diagnosis, it is possible that a larger proportion of patients eventually developed AAU. Additionally, we did not find any statistically significant difference in HLA-B27-positive and HLA-B27-negative children with regard to a family history of HLA-B27-associated disease, which challenges current understanding of the genetic susceptibility of patients to ERA. One possibility is that adult-onset SpA is more greatly affected by one’s genetic milieu and therefore is more common among related family members. Perhaps the development of ERA is influenced less by genetics but more so by environmental factors. Alternatively, given that family history was based on patients’ individual reports, it could be that patients’ knowledge of HLA-B27-associated diseases in their own family history may be limited. Longitudinal studies should further assess the significance of HLA-B27 as a prognostic marker over the natural history of ERA and whether enthesitis follows a different pattern in HLA-B27-negative versus HLA-B27-positive patients.

Our findings should be interpreted in light of several limitations. First, this was a retrospective study spanning several decades from the earliest to the most recent patient. As such, there was not uniform collection of data at the time of initial evaluation, and clinical examinations may have varied between providers and sites. We suspect that treatment practices did vary over time with likely increasing use of TNF-α blockade, but this type of time-series analysis would be best suited as its own separate study. Second, imaging was done at the discretion of the caregiver; there was no uniform screening for hip arthritis or sacroiliitis with imaging. Several reports have shown that sacroiliitis (with sclerotic changes and erosions) is frequently present in children who do not complain of back pain [16, 37, 38]. Therefore, the prevalence of MRI-confirmed sacroiliitis in both groups in this cohort was likely underreported, and our results are likely biased toward the null.

To our knowledge, this study is the largest multicenter international study of children with ERA to date. Both the presenting clinical features and treatment algorithms used for various phenotypes of ERA significantly differed across sites. This heterogeneity in clinical presentation reflects either true differences in patient populations or differences in how the ILAR criteria are being applied. The variation in treatment algorithms emphasizes the need for efficacy and effectiveness studies to help establish evidence-based treatment guidelines tailored to the various clinical phenotypes in this understudied population.

## Conclusions

To our knowledge, this study comprises the largest multicenter international inception cohort of children with ERA to date. Both the presenting clinical features and treatment algorithms used for the various phenotypes of ERA differed significantly across clinical sites. NSAIDs and DMARDs were prescribed as first-line treatments for newly diagnosed ERA, with biologic agents used primarily for sacroiliitis. In comparison with children who were HLA-B27-negative, children who were HLA-B27-positive tended to be older and male and had a higher active joint count, more sacroiliitis, and greater disease activity.

## Abbreviations

AAU: Acute anterior uveitis
ACR: American College of Rheumatology
ANA: Antinuclear antibody
CHAQ: Childhood Health Assessment Questionnaire
cJADAS: clinical Juvenile Arthritis Disease Activity Score
CRP: C-reactive protein
DMARD: Disease-modifying antirheumatic drug
ERA: Enthesitis-related arthritis
ESR: Erythrocyte sedimentation rate
HLA: Human leukocyte antigen
IA: Intra-articular
IBD: Inflammatory bowel disease
IgM: Immunoglobulin M
ILAR: International League of Associations for Rheumatology
IQR: Interquartile range
JIA: Juvenile idiopathic arthritis
JSpADA: Juvenile Spondyloarthritis Disease Activity Index
MRI: Magnetic resonance imaging
NSAID: Nonsteroidal anti-inflammatory drug
RF: Rheumatoid factor
SpA: Spondyloarthritis
TNF-α: Tumor necrosis factor-α
